# Supplementary material for: Selective Oxidation of Glycerol via Acceptorless Dehydrogenation Driven by Ir(I)-NHC Catalysts
Source: Molecules. 2022 Nov 8;27(22):7666. doi: 10.3390/molecules27227666 (PMC9696977; doi:10.3390/molecules27227666)
Supplement: Supplementary file 1 [file molecules-27-07666-s001.zip › molecules-2019978-supplementary.pdf]

Supporting Information

Selective Oxidation of Glycerol via Acceptorless Dehydrogenation Driven by Ir(I)-NHC Catalysts

M. Victoria Jiménez Ana I. Ojeda-Amador, Raquel Puerta-Oteo, Joaquín Martínez-Sal, Vincenzo Passarelli, and Jesús J. Pérez-Torrente**

Departamento de Química Inorgánica, Instituto de Síntesis Química y Catálisis Homogénea-ISQCH, Universidad de Zaragoza-C.S.I.C., 50009-Zaragoza, Spain.

Contents:

1. Selected NMR, IR and HRMS spectra of iridium(I) compounds **1-9**.
2. ^{1}H NMR analysis representative of a catalysis test in the standard reaction conditions.

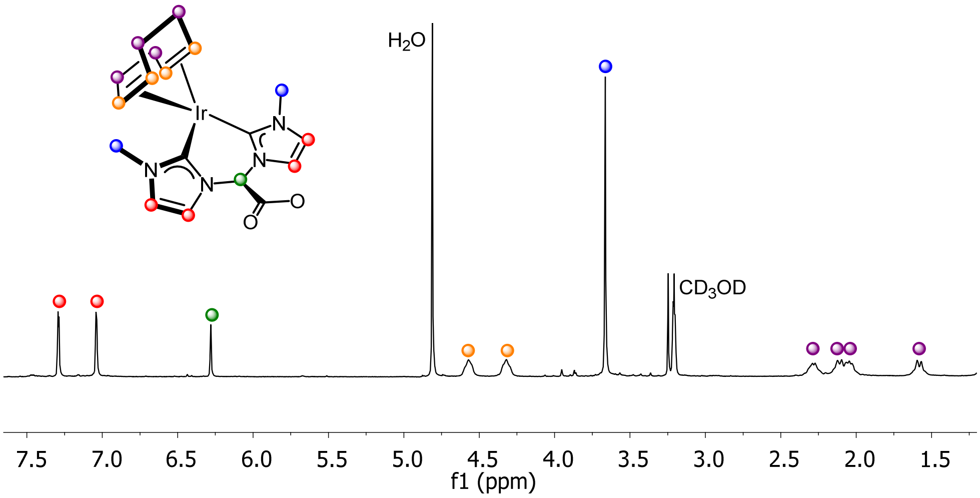

Figure S1. ^{1}H NMR of [Ir(cod){(MeIm)$_2$CHCOO}] (**1**) (CD$_3$OD, 298K).

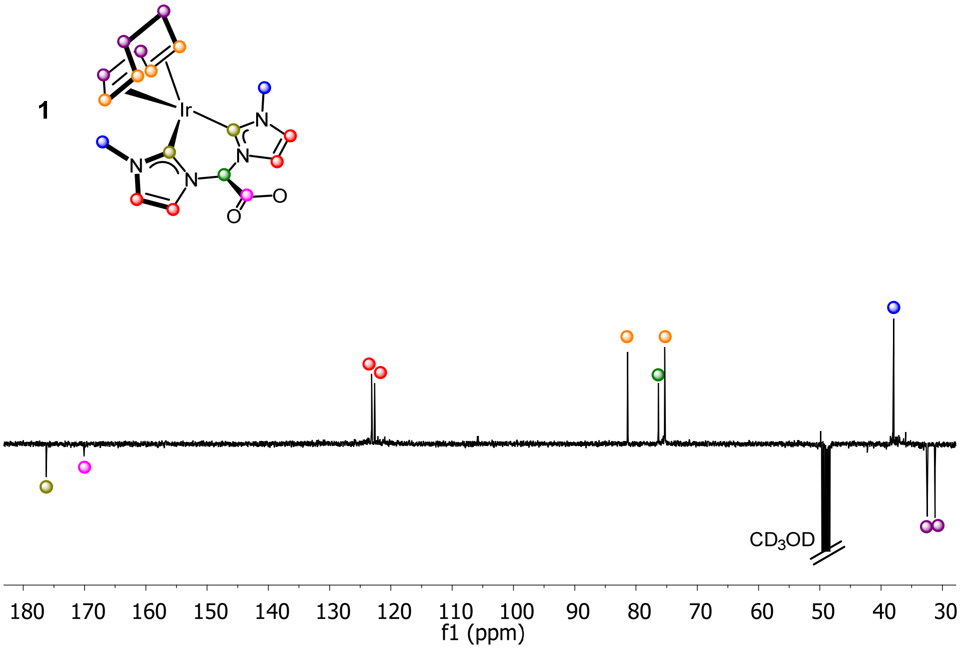

Figure S2. $^{13}C\{^1H\}$-APT NMR of [Ir(cod){(MeIm)$_2$CHCOO}] (**1**) (CD_3OD, 298K).

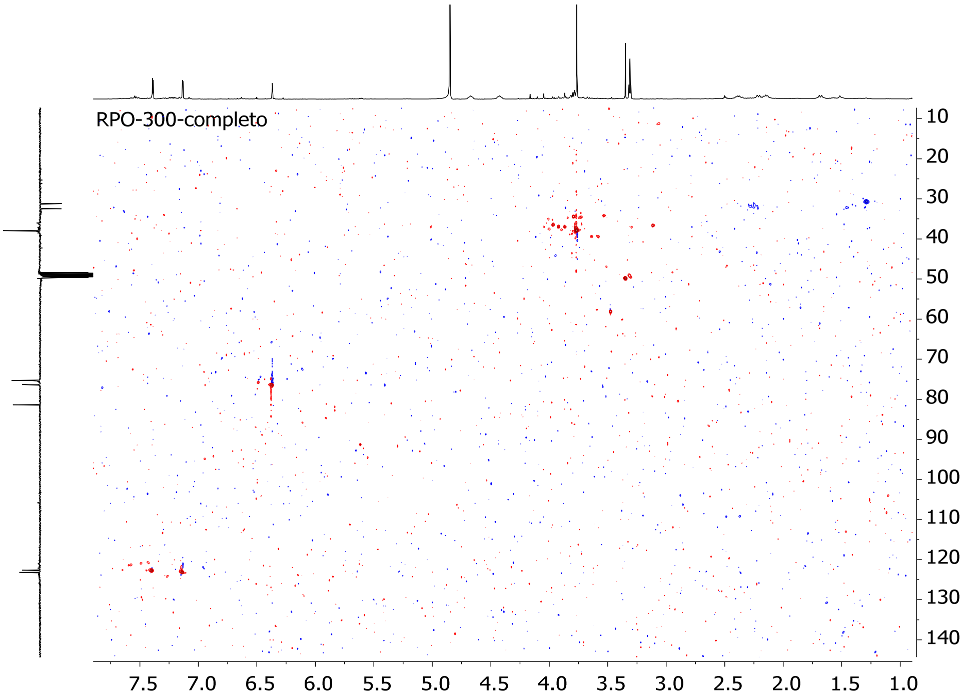

Figure S3. $^1H,^{13}C$-HSQC NMR of [Ir(cod){(MeIm)$_2$CHCOO}] (**1**) (CD_3OD, 298K).

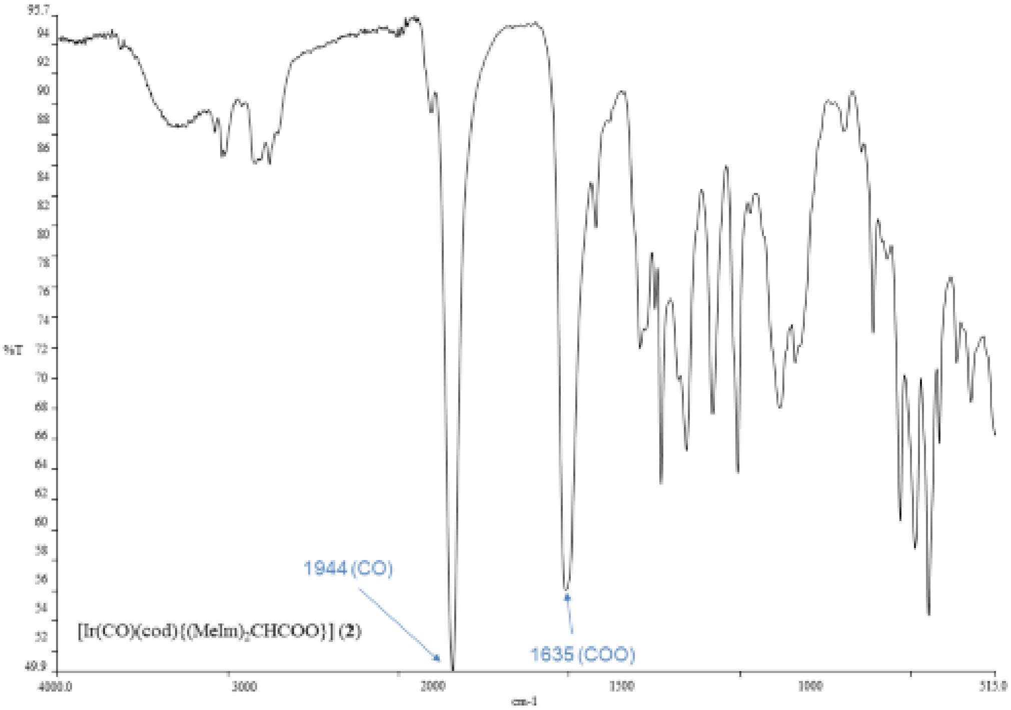

Figure S4. IR spectrum of [Ir(CO)(cod){(MeIm)$_2$CHCOO}] (**2**) (nujol suspension).

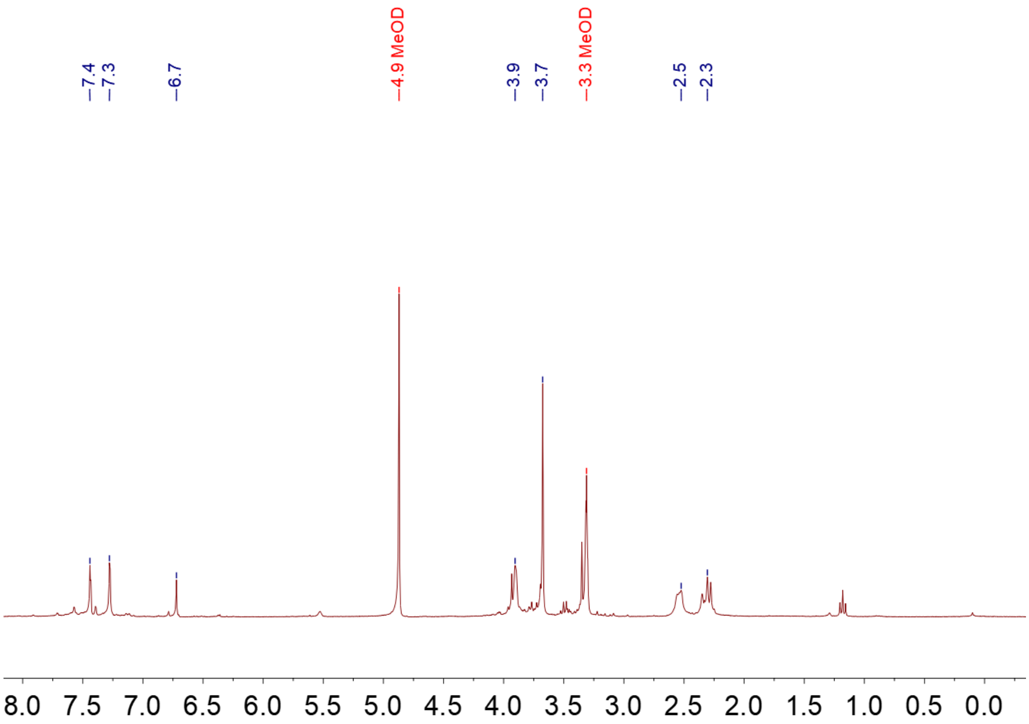

Figure S5. ^{1}H NMR of [Ir(CO)(cod){(MeIm)$_2$CHCOO}] (**2**) (CD_3OD, 298K).

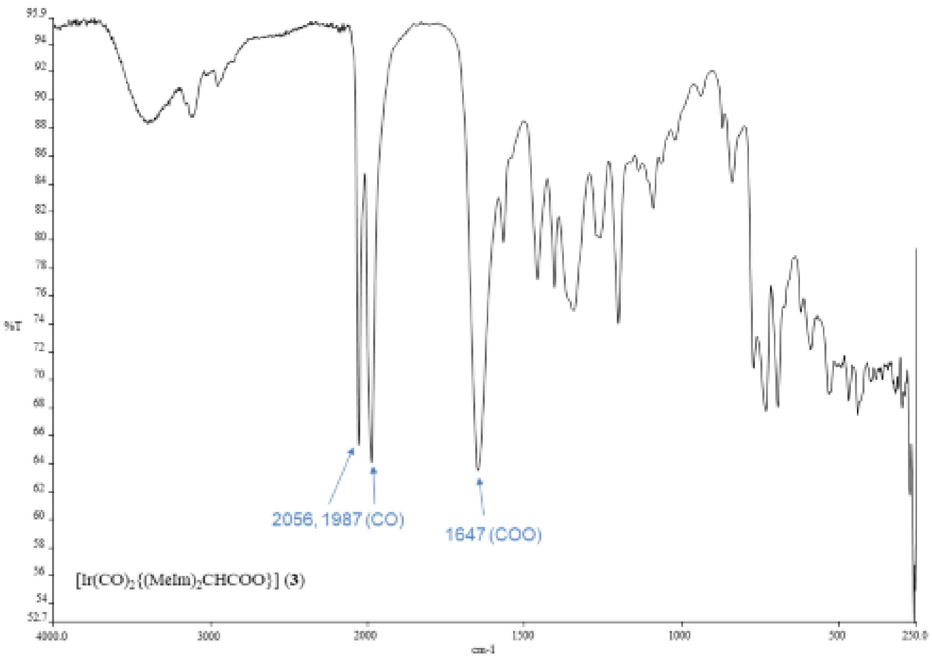

Figure S6. IR spectrum of [Ir(CO)$_2${(MeIm)$_2$CHCOO}] (**3**) (nujol suspension).

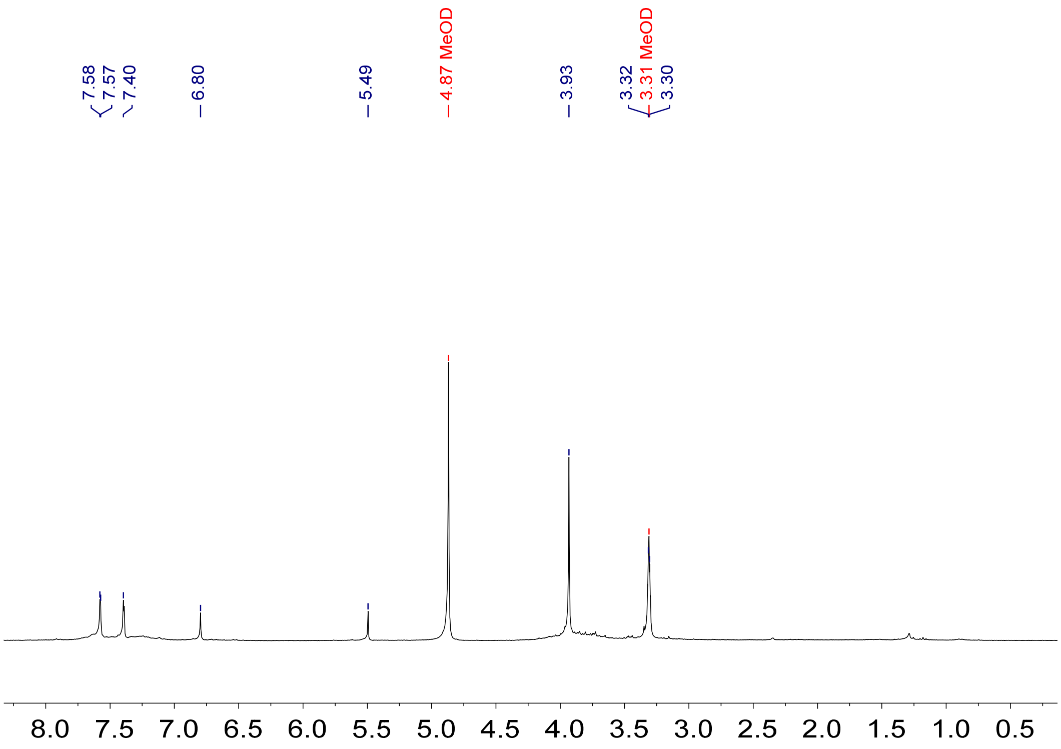

Figure S7. ^{1}H NMR of [Ir(CO)$_2${(MeIm)$_2$CHCOO}] (**3**) (CD_3OD, 298K).

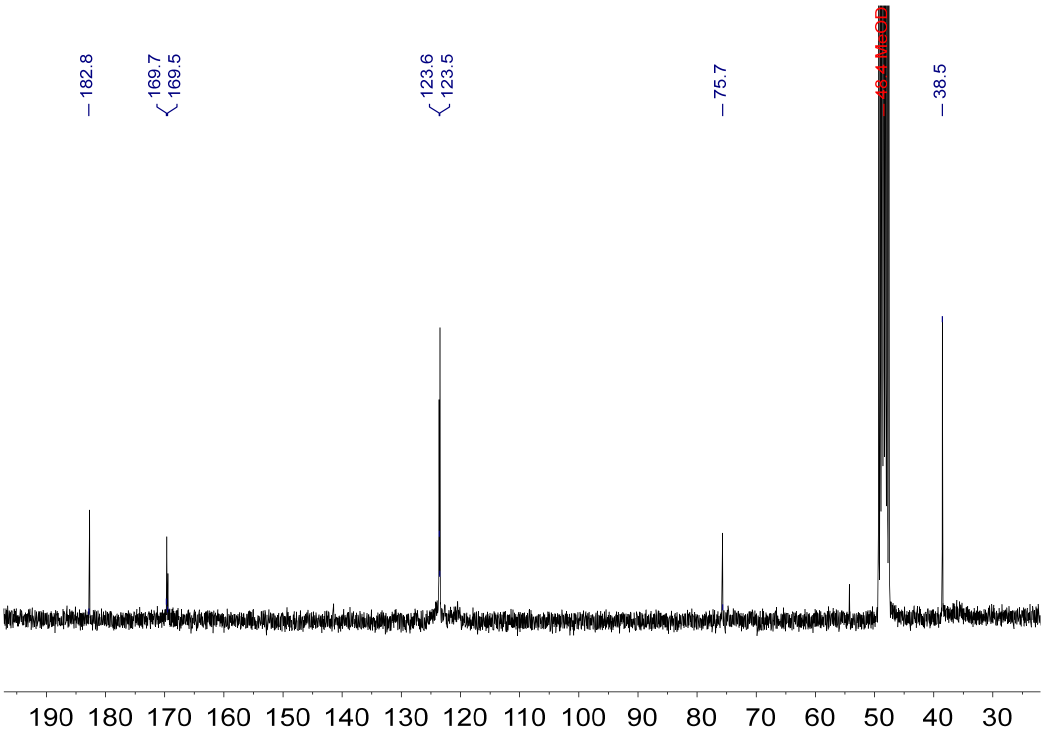

Figure S8. $^{13}C\{^1H\}$ NMR of compound $[Ir(CO)_2\{(MeIm)_2CHCOO\}]$ (**3**) (CD_3OD, 298K).

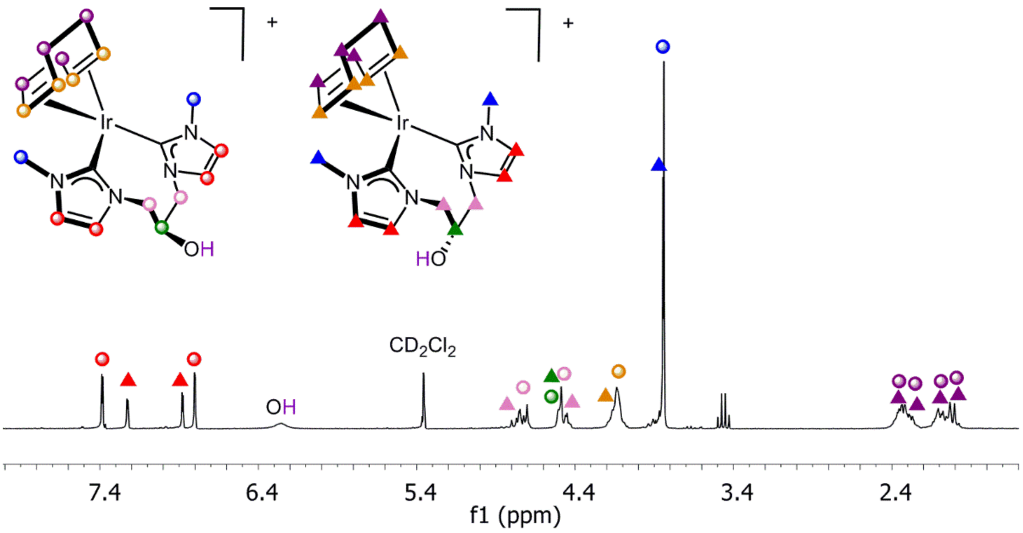

Figure S9. ^1H NMR of $[Ir(cod)\{(MeImCH_2)_2CHOH\}]Br$ (**4**) (CD_2Cl_2, 298K) showing the presence of two isomers.

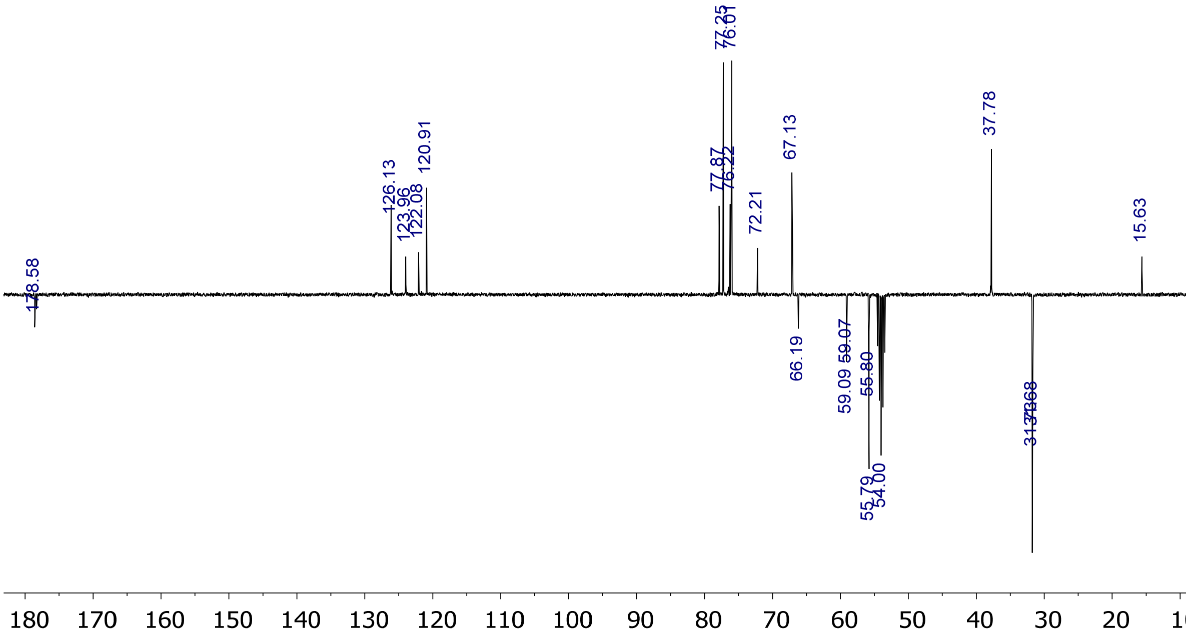

Figure S10. $^{13}C\{^{1}H\}$-APT NMR of [Ir(cod){(MeImCH$_2$)$_2$CHOH}]Br (**4**) (CD_2Cl_2, 298K).

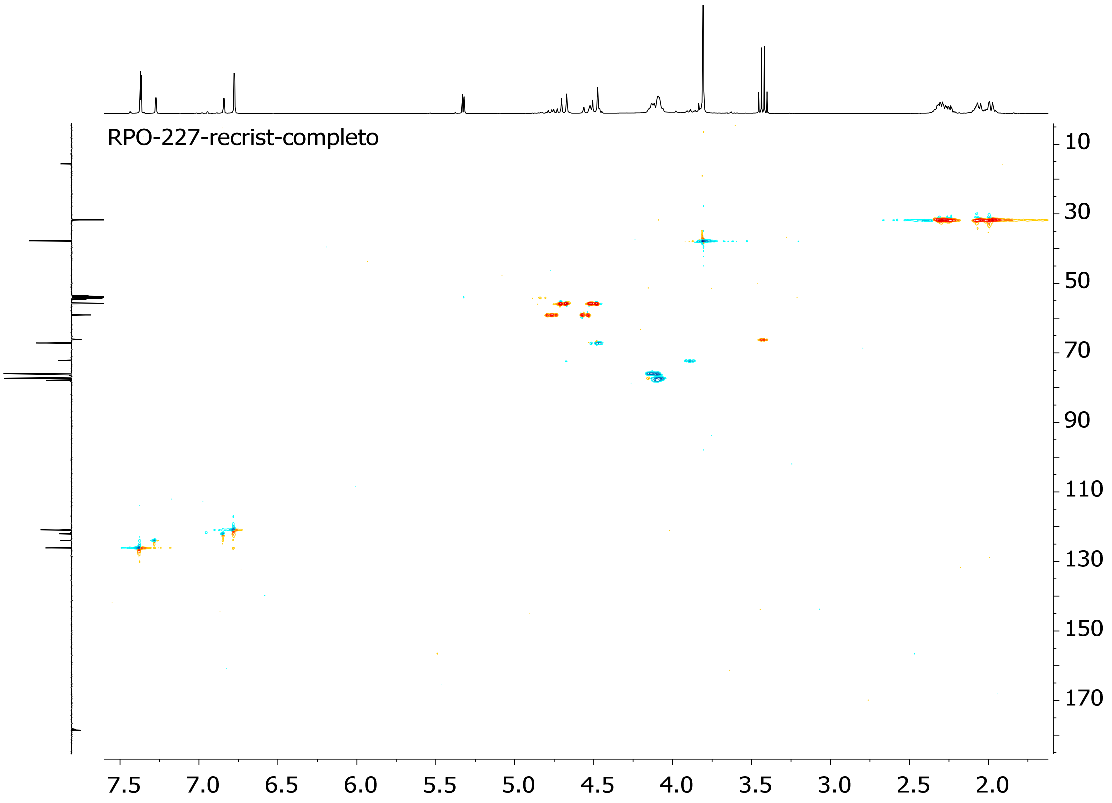

Figure S11. $^{1}H,^{13}C$-HSQC NMR of [Ir(cod){(MeImCH$_2$)$_2$CHOH}]Br (**4**) (CD_2Cl_2, 298K).

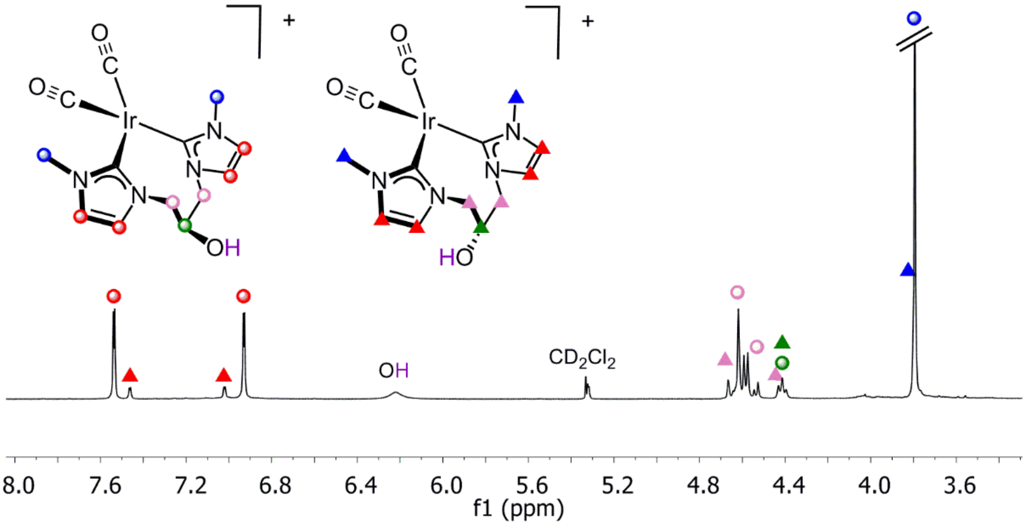

Figure S12. ^{1}H NMR of $[Ir(CO)_2\{(MeImCH_2)_2CHOH\}]Br$ (**5**) (CD_2Cl_2, 298K) showing the presence of two isomers.

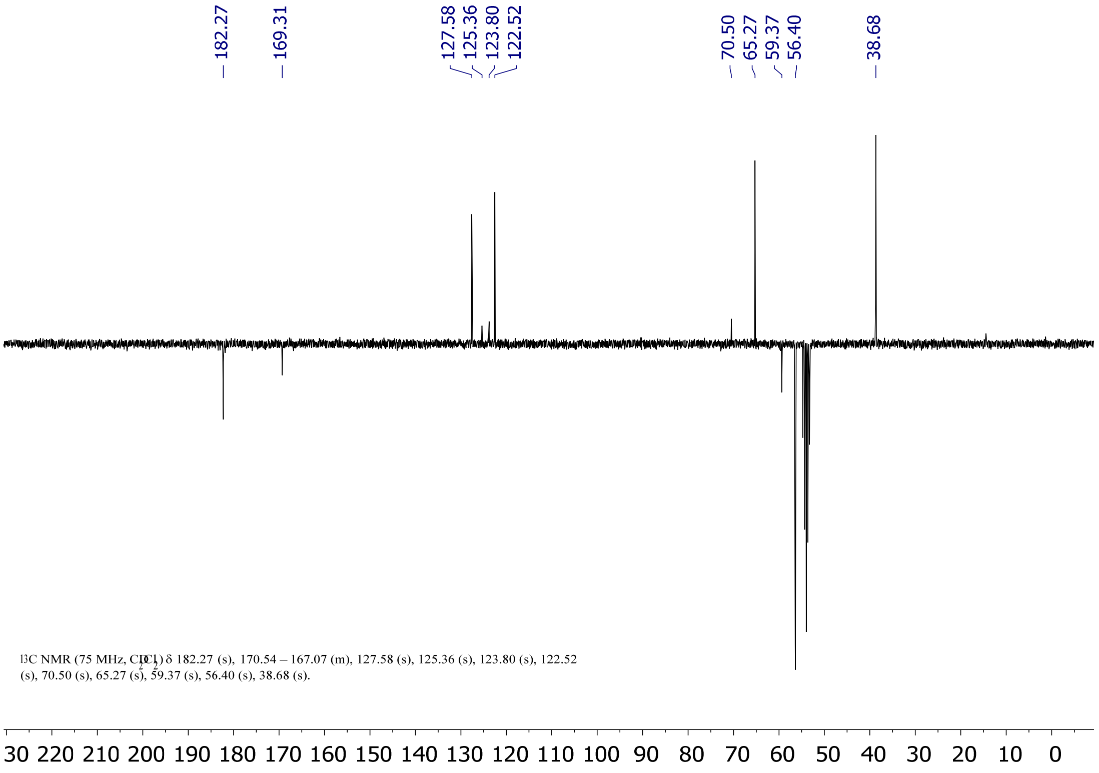

Figure S13. $^{13}C\{^{1}H\}$-APT NMR of $[Ir(CO)_2\{(MeImCH_2)_2CHOH\}]Br$ (**5**) (CD_2Cl_2, 298K).

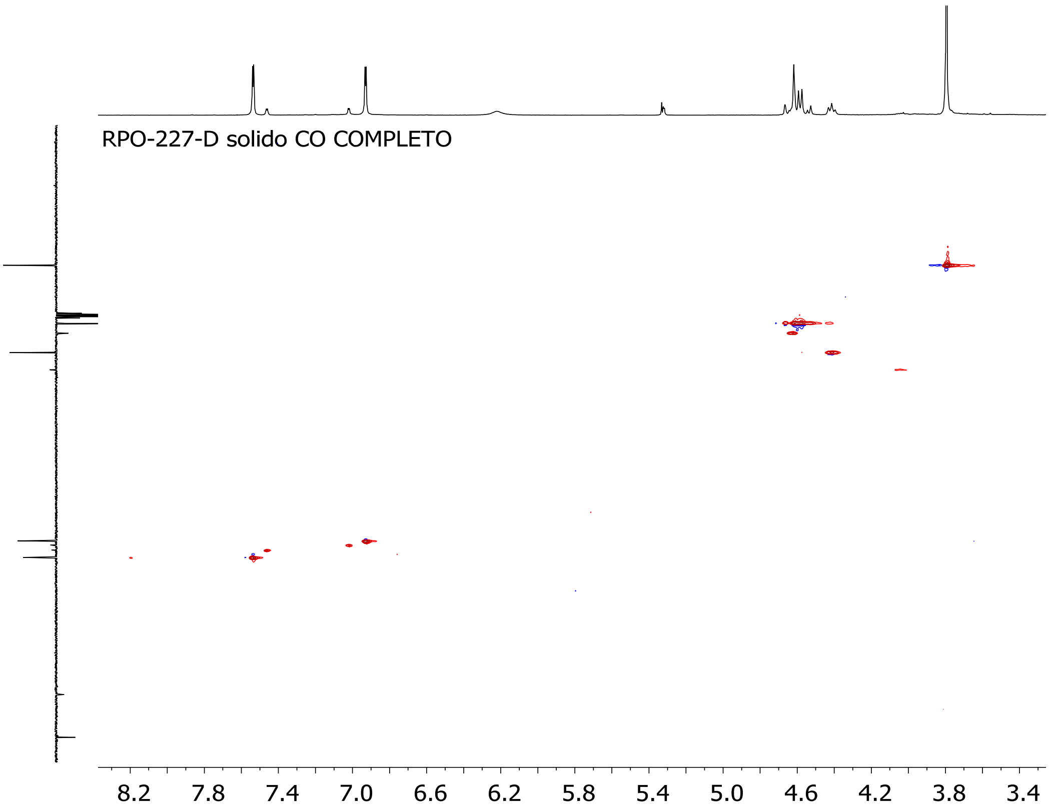

Figure S14. $^{1}H,^{13}C$-HSQC NMR of $[Ir(CO)_2\{(MeImCH_2)_2CHOH\}]Br$ (**5**) (CD_2Cl_2, 298K).

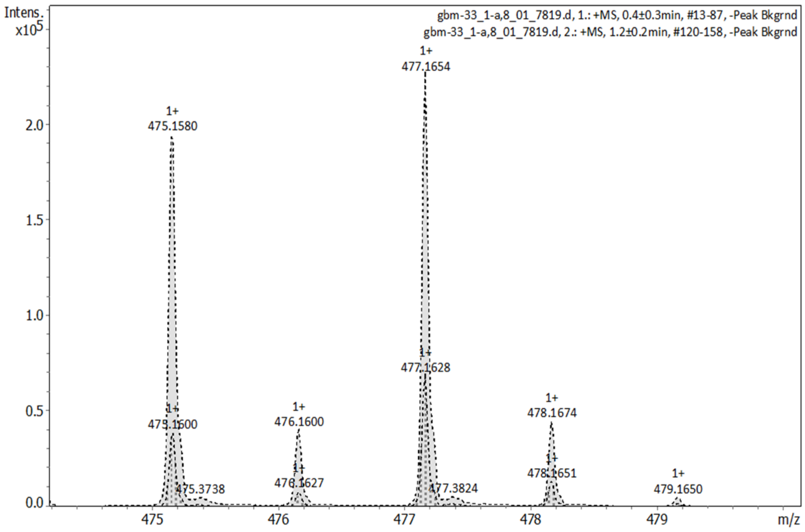

Figure S15. ESI+ mass spectrum of $[Ir(cod)\{(MeIm)_2CH_2\}]I$ (**6**) (CH_2Cl_2).

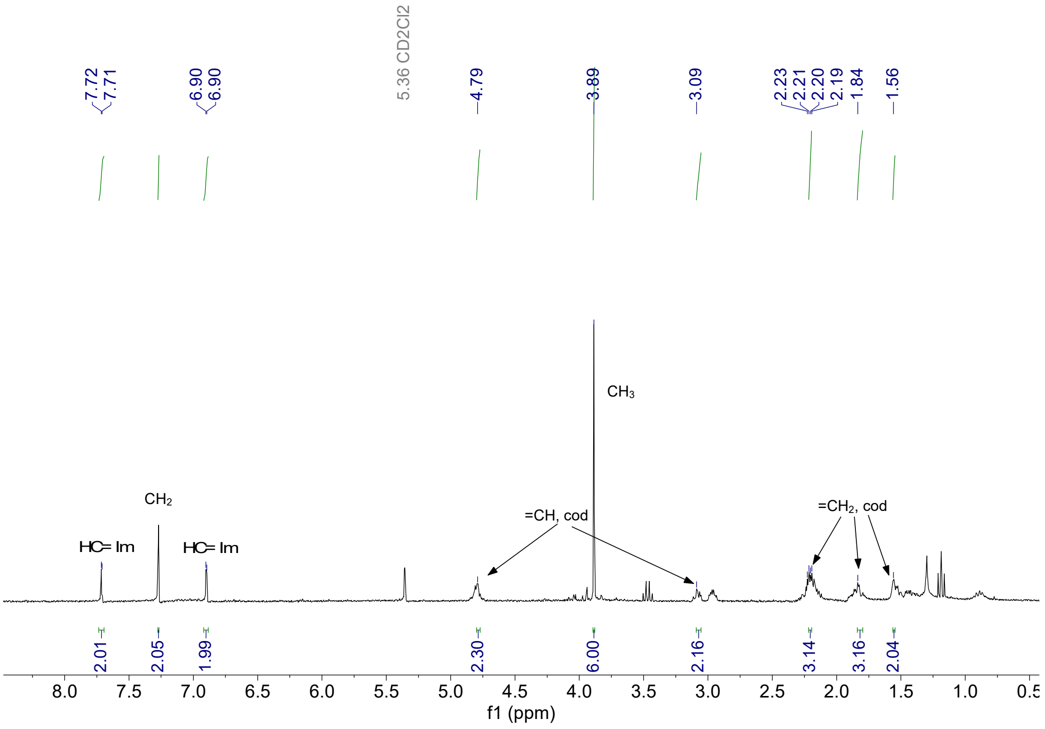

Figure S16. ^{1}H NMR of compound [Ir(cod){(MeIm)$_2$CH$_2$}]I (**6**) (CD_2Cl_2, 298K).

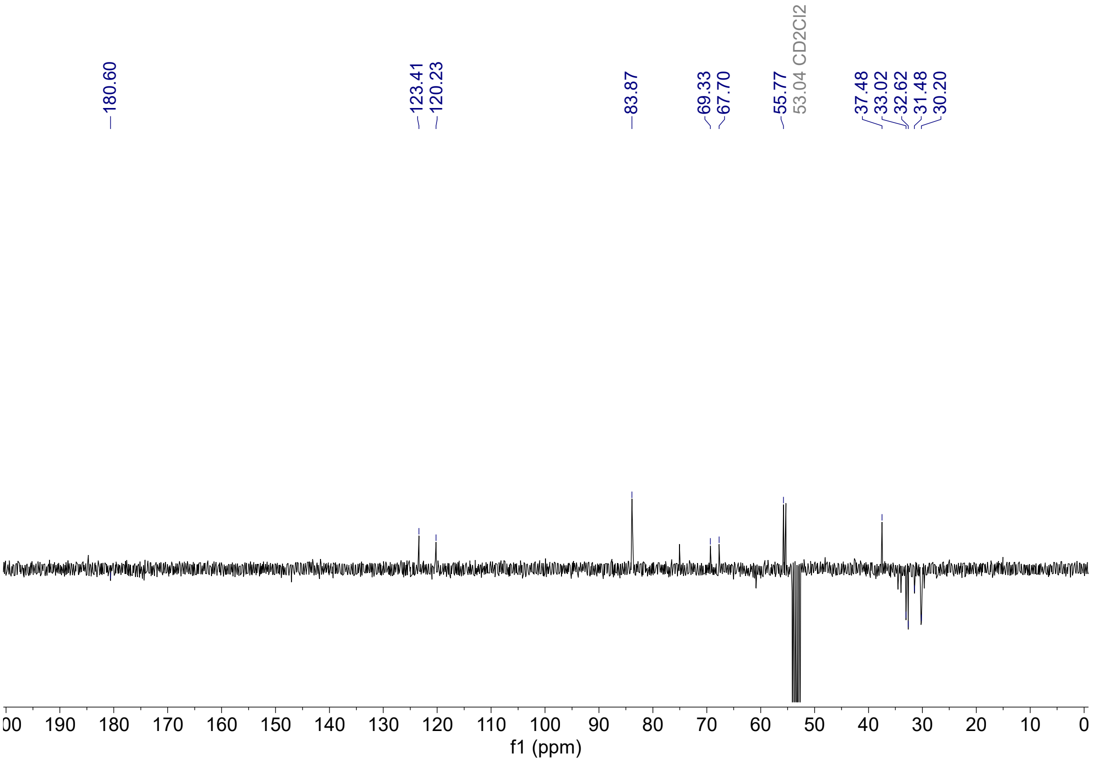

Figure S17. ^{13}C{^{1}H}-APT NMR of [Ir(cod){(MeIm)$_2$CH$_2$}]I (**6**) (CD_2Cl_2, 298K).

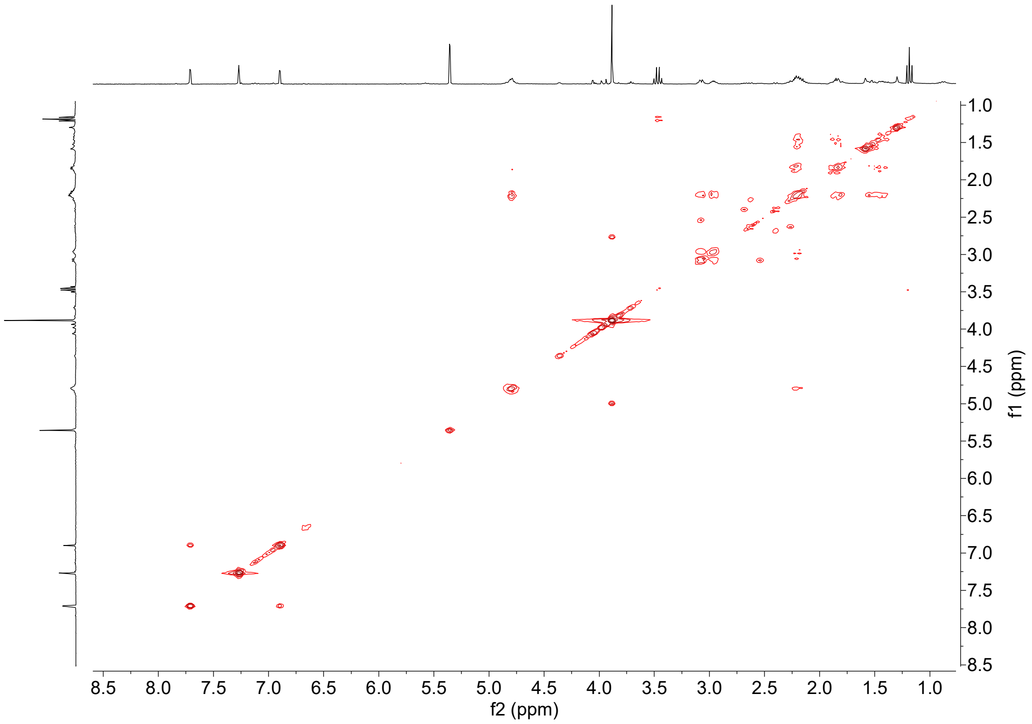

Figure S18. ^{1}H, ^{1}H -COSY NMR of [Ir(cod){$(MeIm)_2CH_2$}]I (**6**) (CD_2Cl_2, 298K).

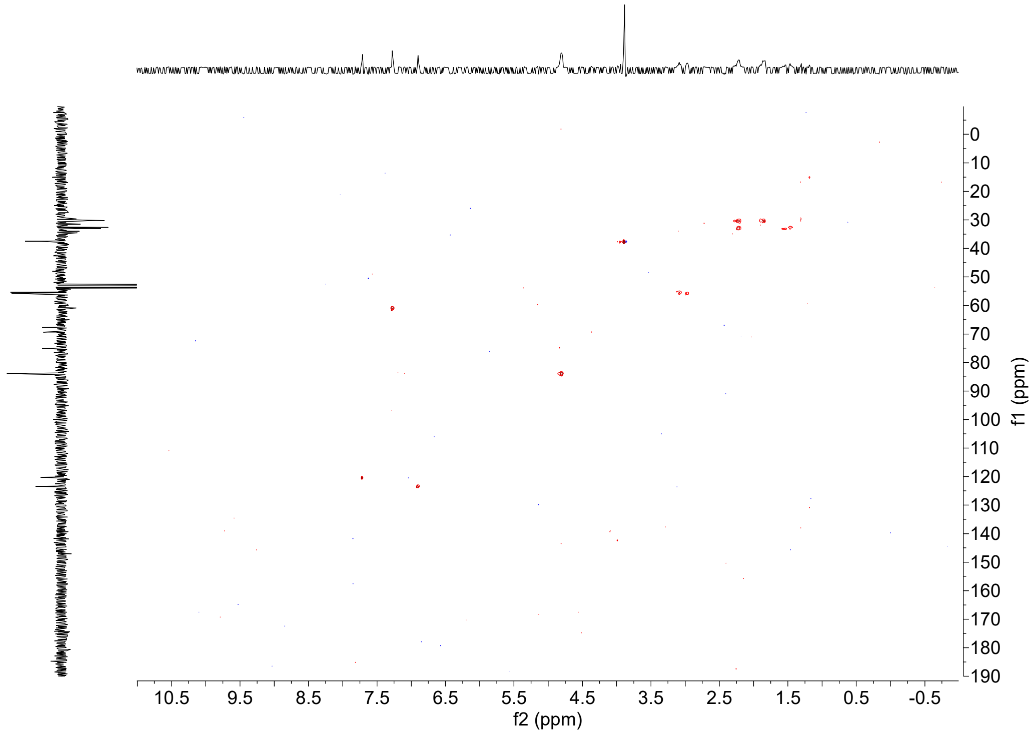

Figure S19. ^{1}H,^{13}C-HSQC NMR of [Ir(cod){$(MeIm)_2CH_2$}]I (**6**) (CD_2Cl_2, 298K).

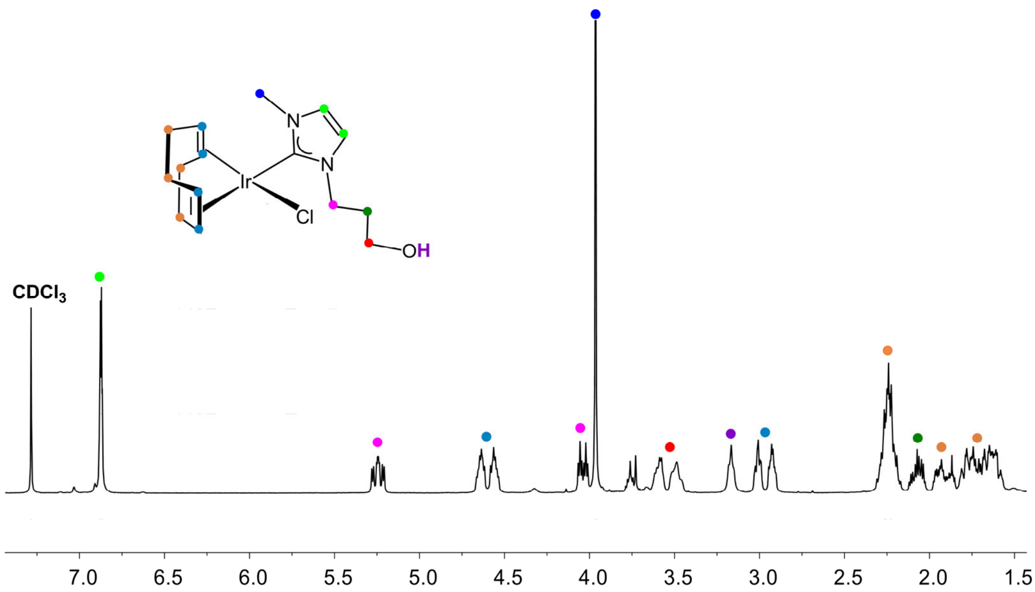

Figure S20. ^{1}H NMR of [IrCl(cod){MeIm(CH$_2$)$_3$OH}] (**7**) (CDCl$_3$, 298K).

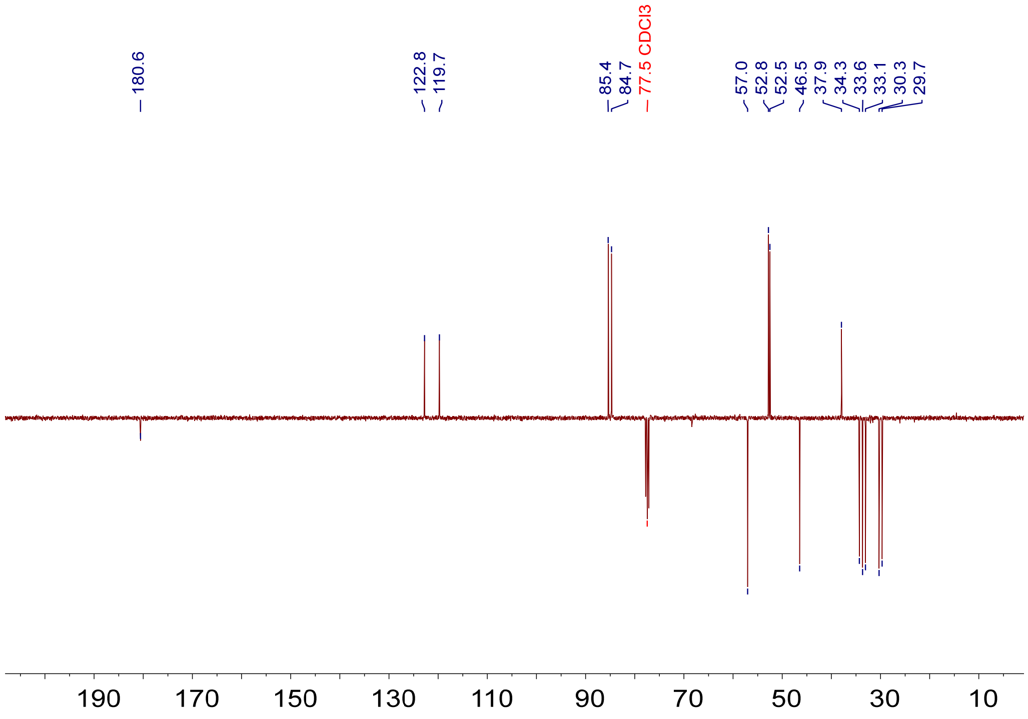

Figure S21. ^{13}C{^{1}H}-APT NMR of [IrCl(cod){MeIm(CH$_2$)$_3$OH}] (**7**) (CDCl$_3$, 298K).

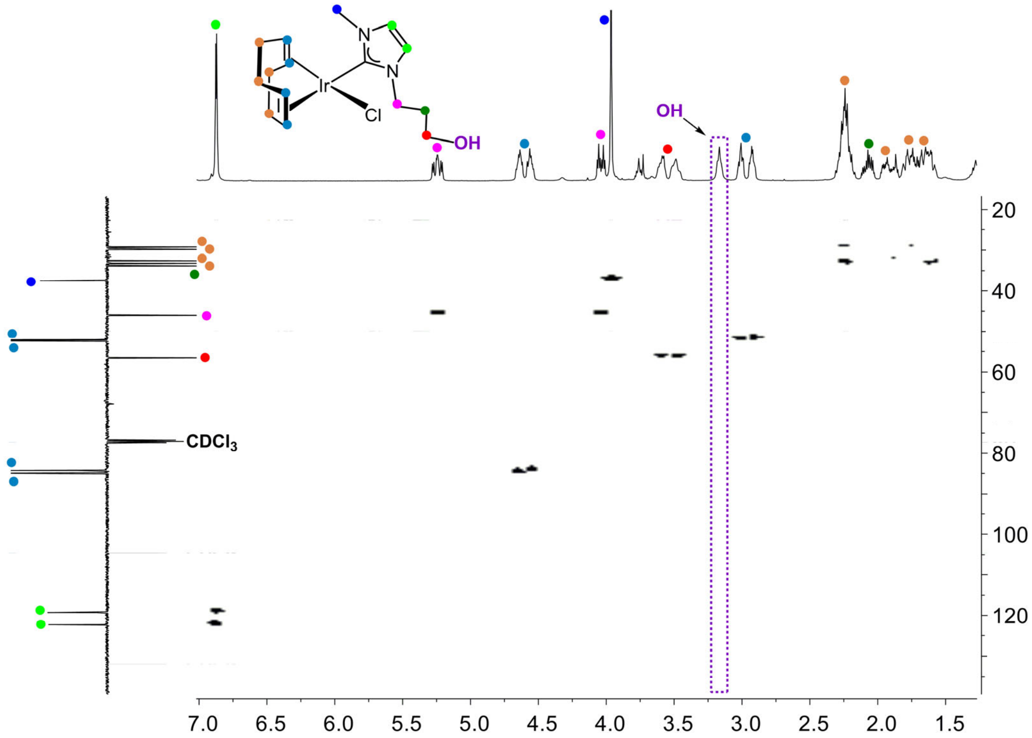

Figure S22. $^{1}H,^{13}C$-HSQC NMR of [IrCl(cod){MeIm(CH$_2$)$_3$OH}] (**7**) (CDCl$_3$, 298K).

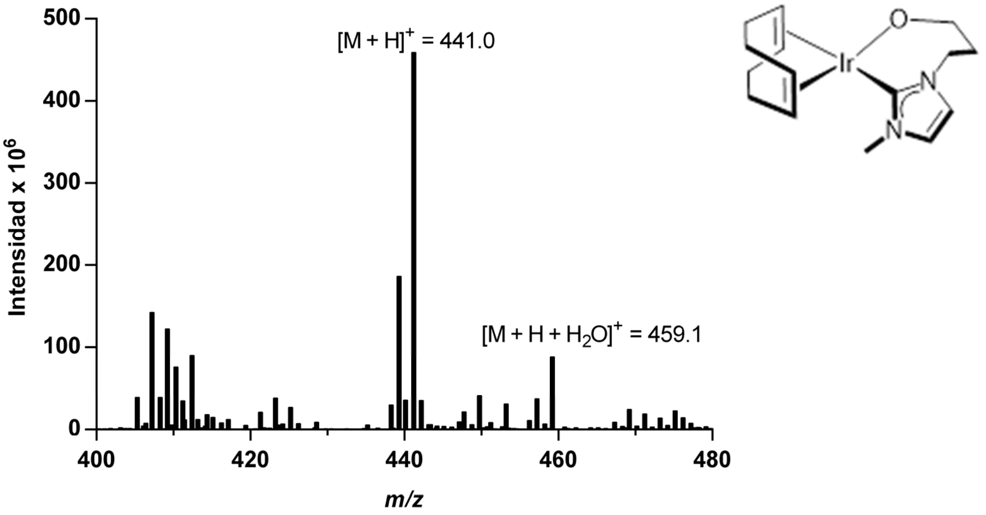

Figure S23. MALDI-Tof mass spectrum of [Ir(cod){κ^2-C,O-{MeIm(CH$_2$)$_3$O}] (**8**) (toluene).

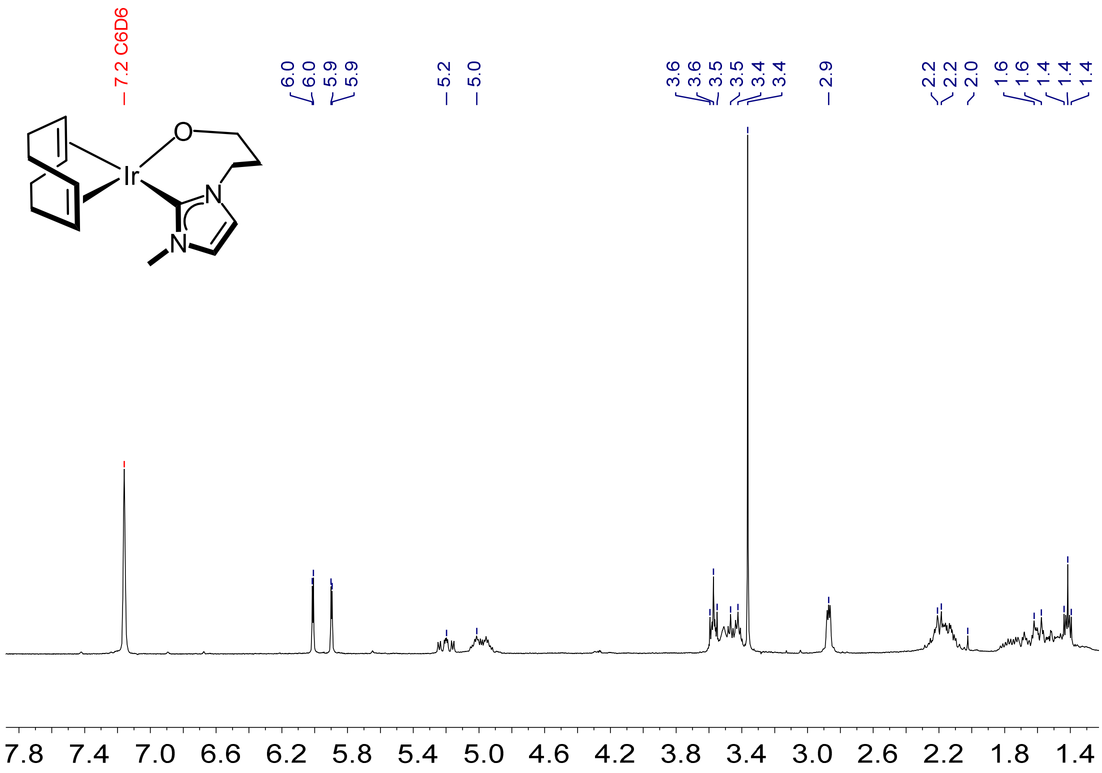

Figure S24. ^{1}H NMR of [Ir(cod){κ^2-C,O-{MeIm(CH_2)$_3$O}] (**8**) (C_6D_6, 298K).

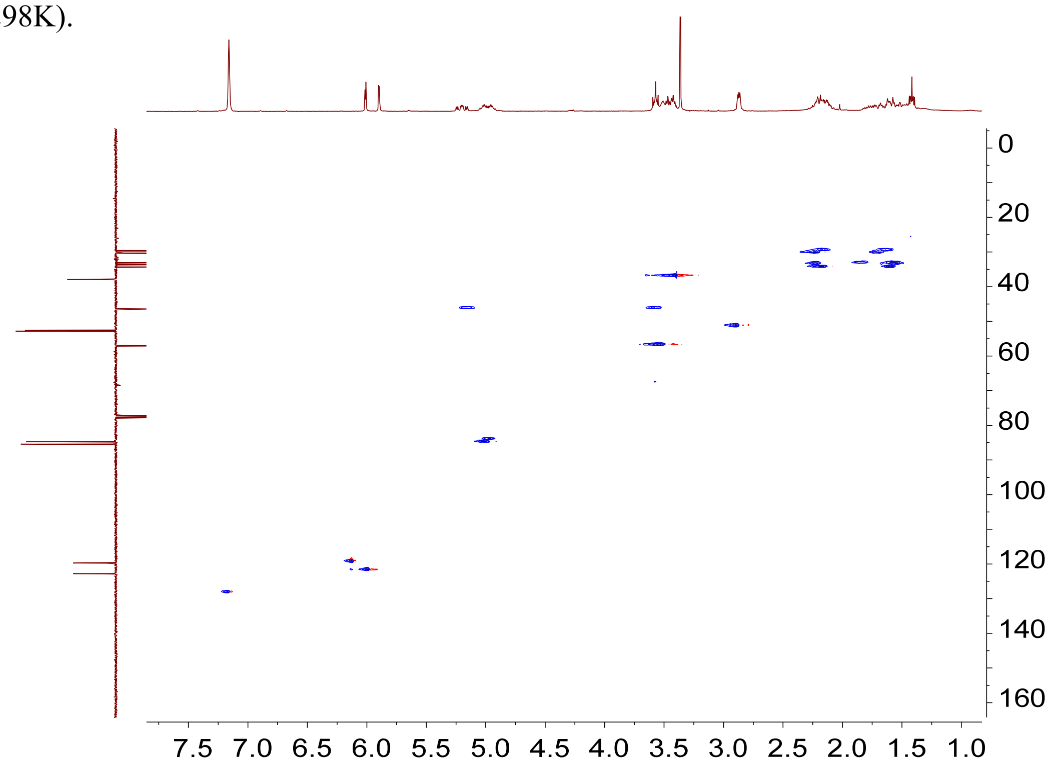

Figure S25. ^{1}H,^{13}C-HSQC NMR of [Ir(cod){κ^2-C,O-{MeIm(CH_2)$_3$O}] (**8**) (C_6D_6, 298K)

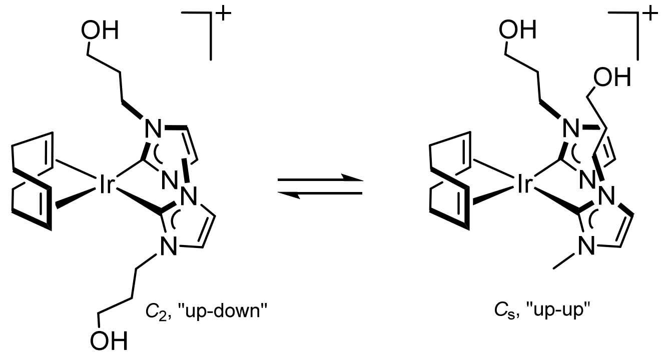

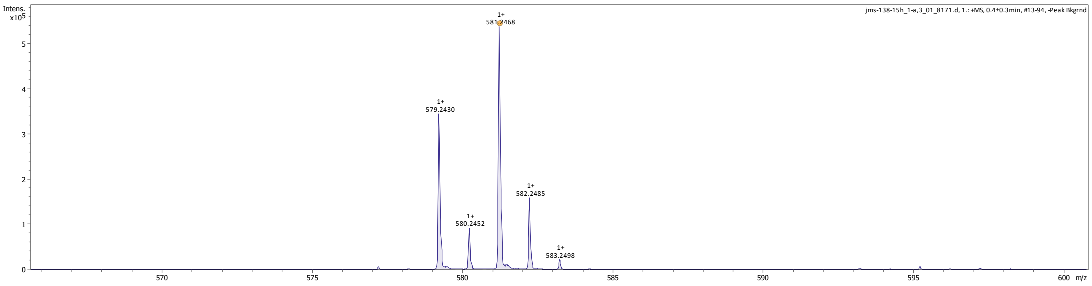

Figure S26. HRMS (ESI^+, MeOH) of compound [Ir(cod){(MeIm(CH₂)₃OH)}₂]Cl (**9**), *m/z* = 581.2468 $[M]^+$.

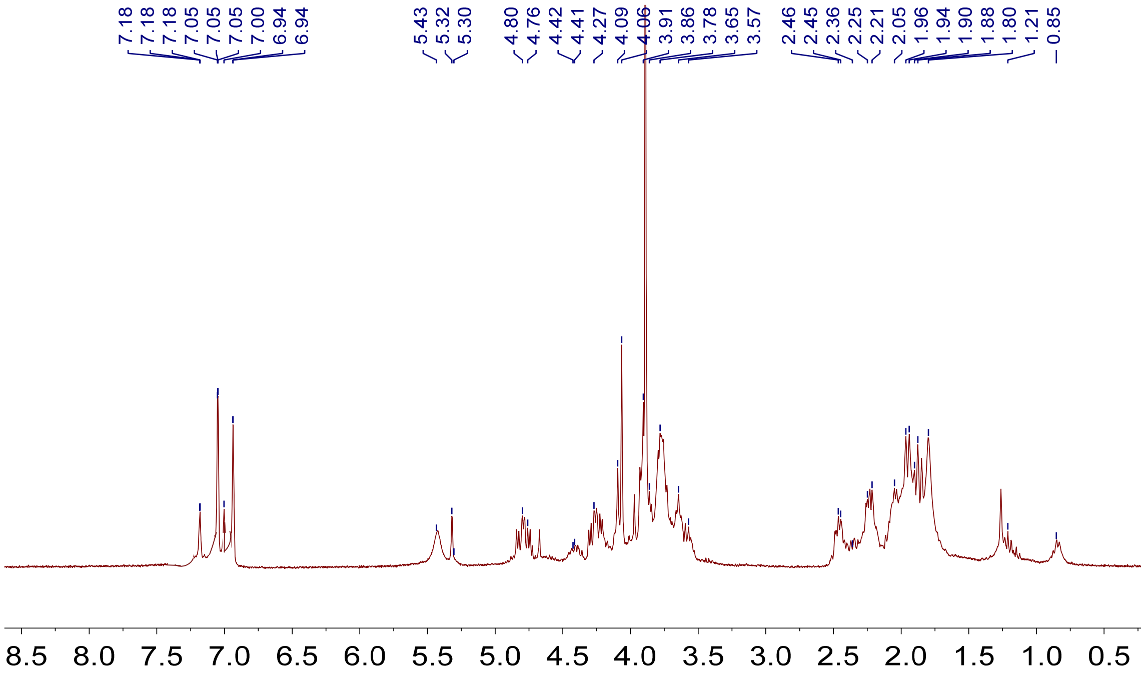

Figure S27. ^{1}H NMR of [Ir (cod){(MeIm(CH₂)₃OH)}₂]Cl (**9**) (CD_2Cl_2, 298K).

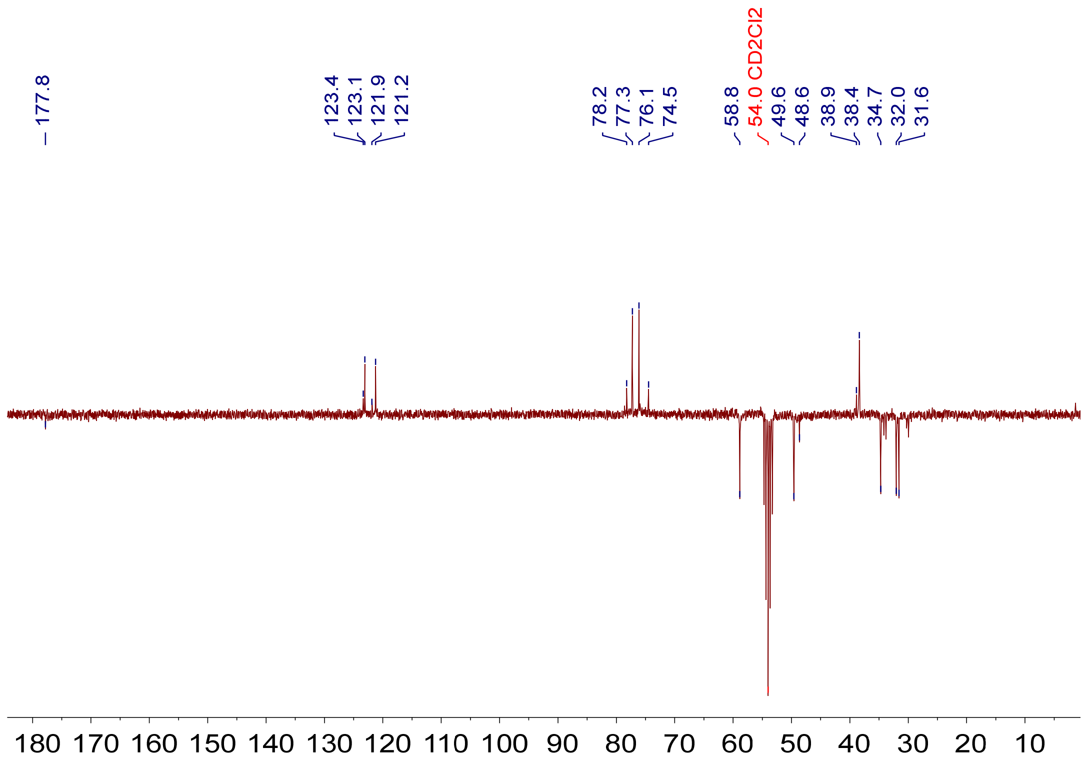

Figure S28. $^{13}C\{^{1}H\}$-APT NMR of [Ir (cod){(MeIm(CH$_2$)$_3$OH)}$_2$]Cl (**9**) (CD_2Cl_2, 298K).

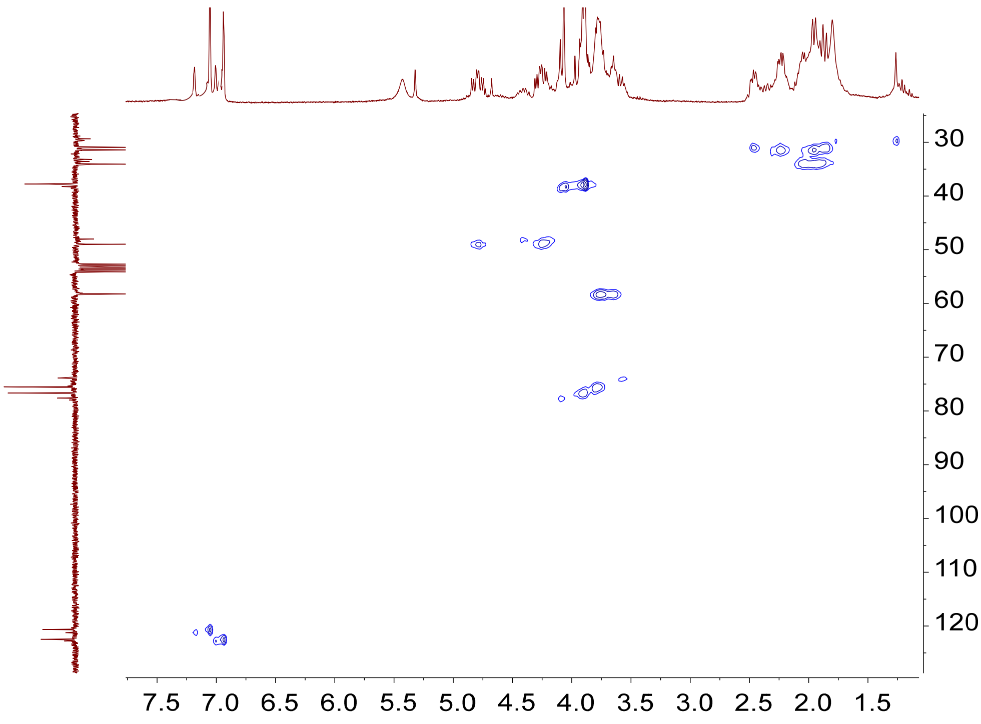

Figure S29. $^{1}H,^{13}C$-HSQC NMR of [Ir (cod){(MeIm(CH$_2$)$_3$OH)}$_2$]Cl (**9**) (CD_2Cl_2, 298K).

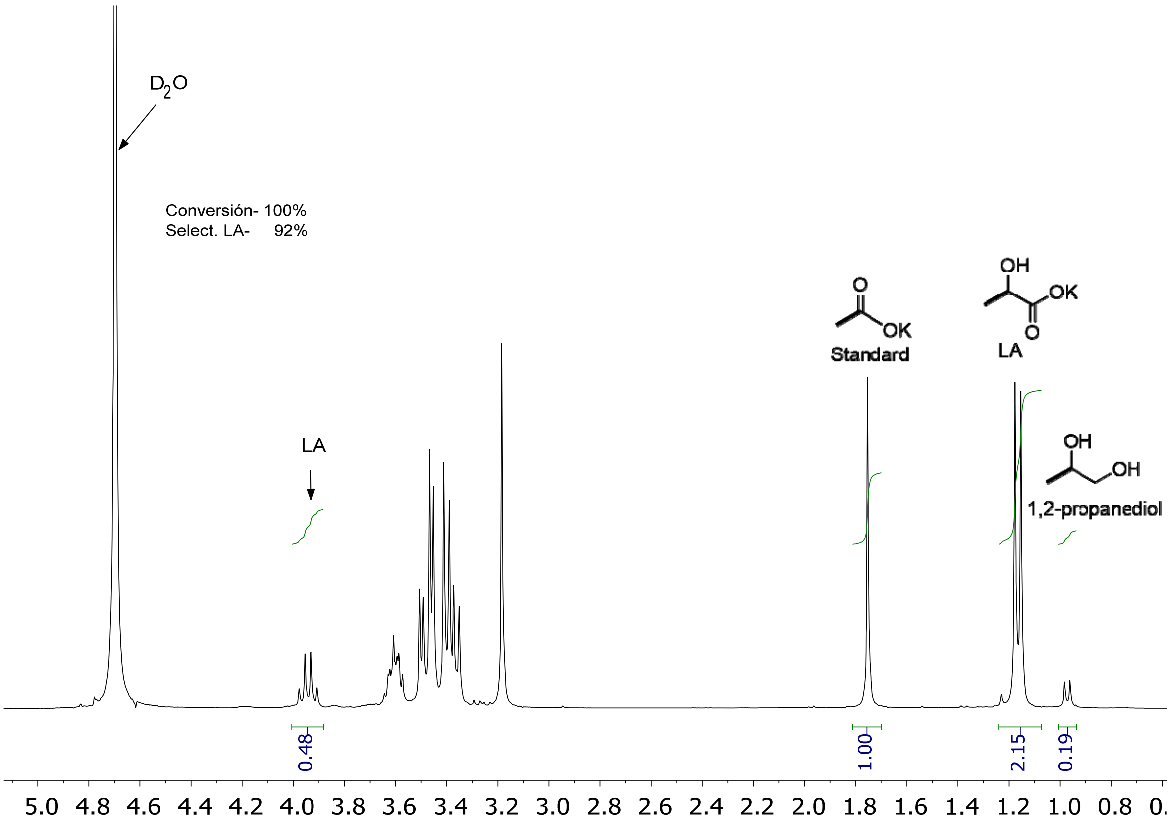

Figure S30. ^{1}H NMR (D_2O, 298K) using NaOAc as internal standard for a catalysis test in the standard reaction conditions: glycerol (1 mL, 13.7 mmol), KOH (5 mmol) and iridium catalyst (0.2 mmol%). Conversion of glycerol, based on the theoretical maximum determined by the mmol of added base, potassium lactate (LA).
